# Unique long non-coding RNA expression signature in ETV6/RUNX1-driven B-cell precursor acute lymphoblastic leukemia

**DOI:** 10.18632/oncotarget.12063

**Published:** 2016-09-16

**Authors:** Farzaneh Ghazavi, Barbara De Moerloose, Wouter Van Loocke, Annelynn Wallaert, Hetty H. Helsmoortel, Alina Ferster, Marleen Bakkus, Geneviève Plat, Eric Delabesse, Anne Uyttebroeck, Filip Van Nieuwerburgh, Dieter Deforce, Nadine Van Roy, Frank Speleman, Yves Benoit, Tim Lammens, Pieter Van Vlierberghe

**Affiliations:** ^1^ Department of Pediatric Hematology-Oncology and Stem Cell Transplantation, Ghent University Hospital, Ghent, Belgium; ^2^ Center for Medical Genetics, Department of Paediatrics and Genetics, Ghent University Hospital, Ghent, Belgium; ^3^ Department of Hemato-Oncology, HUDERF, Université Libre de Bruxelles (ULB), Brussels, Belgium; ^4^ Department of Hematology, University Hospital Brussels, Vrije Universiteit Brussel (VUB) Brussels, Belgium; ^5^ Department of Hematology, Children's Hospital, Toulouse, France; ^6^ Department of Hematology, Institut Universitaire de Cancérologie de Toulouse, University Toulouse-III Paul-Sabatier, Toulouse, France; ^7^ Department of Pediatric Hemato-Oncology, University Hospitals Leuven, Belgium; ^8^ Laboratory of Pharmaceutical Biotechnology, Department of Pharmaceutics, Ghent University, Ghent, Belgium

**Keywords:** BCP-ALL, ETV6/RUNX1, LncRNA

## Abstract

Overwhelming evidence indicates that long non-coding RNAs have essential roles in tumorigenesis. Nevertheless, their role in the molecular pathogenesis of pediatric B-cell precursor acute lymphoblastic leukemia has not been extensively explored. Here, we conducted a comprehensive analysis of the long non-coding RNA transcriptome in *ETV6/RUNX1-positive* BCP-ALL, one of the most frequent subtypes of pediatric leukemia. First, we used primary leukemia patient samples to identify an ETV6/RUNX1 specific expression signature consisting of 596 lncRNA transcripts. Next, integration of this lncRNA signature with RNA sequencing of BCP-ALL cell lines and lncRNA profiling of an *in vitro* model system of *ETV6/RUNX1* knockdown, revealed that *lnc-NKX2-3-1*, *lnc-TIMM21-5, lnc-ASTN1-1* and *lnc-RTN4R-1* are truly regulated by the oncogenic fusion protein. Moreover, sustained inactivation of *lnc-RTN4R-1* and *lnc-NKX2-3-1* in ETV6/RUNX1 positive cells caused profound changes in gene expression. All together, our study defined a unique lncRNA expression signature associated with *ETV6/RUNX1-positive* BCP-ALL and identified *lnc-RTN4R-1* and *lnc-NKX2-3-1* as lncRNAs that might be functionally implicated in the biology of this prevalent subtype of human leukemia.

## INTRODUCTION

Acute lymphoblastic leukemia (ALL) is an aggressive hematological malignancy that results from the accumulation of genetic alterations in B- or T-lymphoid precursor cells [1–3]. Approximately 25% of pediatric B-cell precursor ALL (BCP-ALL) patients carry the t(12;21)(p13;q22) translocation, which fuses the N-terminal part of the *ETS* variant 6 (*ETV6*) with virtually the entire Runt-related transcription factor 1 (*RUNX1*). Although most *ETV6/RUNX1-positive* BCP-ALL patients have an excellent prognosis with current treatment protocols, some patients still experience a late disease relapse [4, 5].

Ample evidence, including the detection of ETV6/RUNX1 fusion transcripts in neonatal blood spots as well as the development of concordant B-ALL in monozygotic twins, has suggested that t(12;21)(p13;q22) translocation can originate *in utero* [6]. Nevertheless, several additional genetic alterations are supposed to accumulate in *ETV6/RUNX1-positive* pre-leukemic hematopoietic cells in order to trigger full malignant transformation [7].

Previous reports have shown that the ETV6/RUNX1 fusion protein leads to expansion of B-cell precursors with enhanced self-renewal capacity and impaired differentiation [8–13]. In addition, several mechanisms of action have been proposed for this chimeric protein, including repression of normal RUNX1 target genes, disruption of wild type ETV6 transcriptional activity and re-localization of transcriptional cofactors from the nucleus into the cytoplasm [14–16]. Finally, genome-wide profiling studies have shown that *ETV6/RUNX1*-positive leukemias are characterized by a unique gene expression signature as compared to other genetic subtypes of human BCP-ALL with differential expression of genes involved in differentiation, apoptosis, signal transduction and immune response [17–19].

Recently, it became clear that a significant portion of the human genome consists of non-coding transcripts such as tRNAs, snoRNAs, microRNAs and long non-coding RNAs (lncRNAs). Although only a fraction of lncRNAs have been functionally characterized, increasing evidence shows that these long non-coding transcripts can play important roles in different cellular processes, including regulation of gene expression, guidance of chromatin remodeling complexes, X-chromosome inactivation, genomic imprinting, nuclear compartmentalization, nuclear cytoplasmic trafficking, RNA splicing and translational control. Moreover, besides their putative roles in normal development, lncRNAs have also been implicated in a variety of human diseases, including cancer [20–23]. Sporadic examples of lncRNAs involved in the pathogenesis of BCP-ALL have been reported. For example, overexpression of lncRNA *BALR-2* correlated with poor clinical outcome and a reduced prednisone response in human BCP-ALLs [24]. Furthermore, another study identified a unique lncRNA expression signature in MLL-rearranged ALLs, which could be exploited for the identification of novel biomarkers for this disease [25]. Nevertheless, the full spectrum of lncRNA expression patterns in BCP-ALL remains largely unexplored.

Here, we provide a comprehensive overview of lncRNA expression in *ETV6/RUNX1*-positive BCP-ALL and analyze the transcriptional consequences of lncRNA modulation in the context of *ETV6/RUNX1* rearranged leukemia.

## RESULTS

### LncRNA expression in ETV6/RUNX1-driven BCP-ALL

**Figure 1:**
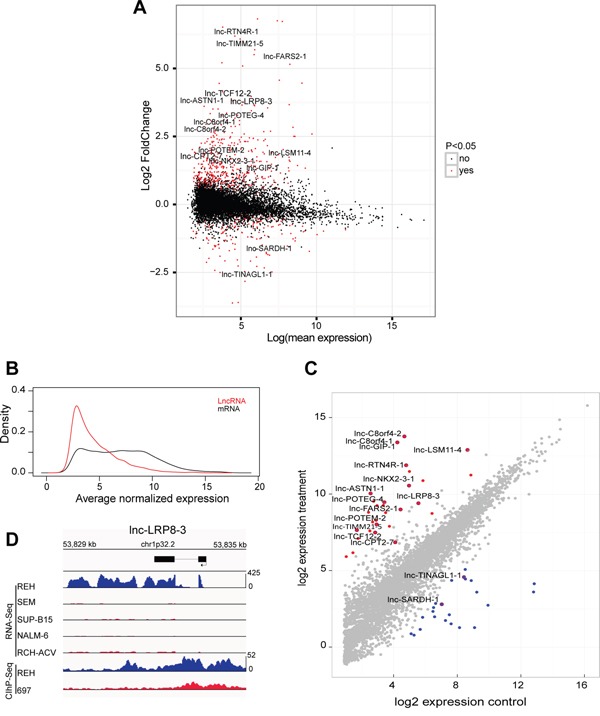
Specific lncRNA expression pattern in ETV6/RUNX1-driven BCP-ALL **A.** MA-plots, which display the mean expression and fold change of differentially expressed lncRNAs in ETV6/RUNX1 driven leukemias (adjusted p-value < 0.05) identified by microarray expression profiling **B.** Density plot of average normalized expression values for lncRNAs and mRNA transcripts in 25 *ETV6/RUNX1*-positive BCP-ALLs **C.** Diagonal plot that represents differentially up-regulated (Red) and down-regulated (Blue) lncRNAs in an *ETV6/RUNX1*-positive BCP-ALL cell line identified by RNA-sequencing of 13 BCP-ALL cell lines (adjusted p-value < 0.05). **D.** RNA sequencing tracks that represent expression of lnc-LRP8-3 in five BCP-ALL cell lines and H3K27ac binding patterns (ChIP-Seq) of the same genomic locus in REH and 697 cells.

To study the role of lncRNAs in the molecular pathogenesis of human BCP-ALL, we profiled a cohort of 64 primary BCP-ALL patient samples using a previously published microarray platform that simultaneously detects lncRNA and mRNA transcripts [26]. This patient cohort consisted of 25 *ETV6/RUNX1*-positive, 7 *TCF3/PBX1*-positive, 15 high hyperdiploid and 17 normal karyotype BCP-ALLs. Using 14055 probes that showed expression above the background, we established an ETV6/RUNX1-specific lncRNA signature consisting of 596 lncRNA transcripts (434 up- and 162 down-regulated) that showed significant differential expression between *ETV6/RUNX1*-positive BCP-ALL and other genetic BCP-ALL subclasses (adj. p-value<0.05, Figure 1A, Supplementary Table S1). In our dataset, average lncRNA expression was lower as compared to mRNA transcripts (Figure 1B), a notion that has previously been reported in other studies [27].

### Identification of ETV6/RUNX1 specific lncRNAs by RNA sequencing of human BCP-ALL cell lines

To further validate our initial findings and to evaluate the *ETV6/RUNX1*-positive BCP-ALL cell line REH, as a suitable *in vitro* model system to study the functional relevance of *ETV6/RUNX1*-specific lncRNAs, we analyzed publically available non-stranded poly-A RNA sequencing data from 13 human BCP-ALL tumor lines including REH and 12 other non-*ETV6/RUNX1* cell lines (SEM, SUP-B15, NALM-6, NALM-19, RCH-ACV, RS4;11, KASUMI-2, KOPN-8, MUTZ-5, MHH-CALL-2, MHH-CALL-3 and MHH-CALL-4).

More specifically, using differential expression analysis of sequence count data (DESeq2 [28]), we identified 50 lncRNAs that showed a significant differential expression between REH and the other non-*ETV6/RUNX1* cell lines (Generalized Linear Model; adj. p-value <0.05), including 28 up- and 22 down-regulated lncRNA transcripts (Figure 1C; Supplementary Table S2). Interestingly, 16 out of 50 lncRNAs overlapped with the *ETV6/RUNX1*-specific lncRNA signature that was identified in the primary leukemia samples, including 14 up- and 2 down-regulated lncRNA transcripts (Figure 1C; Supplementary Table S3).

Next, we performed chromatin immunoprecipitation followed by sequencing (ChIP-seq) to evaluate the genome-wide distribution of H3K27 acetylation (H3K27ac), a histone mark indicative for active promoters and/or putative enhancer regions, in an *ETV6/RUNX1*-positive (REH) and negative (697) BCP-ALL cell line. Notably, differential H3K27ac binding between REH and 697 cells was identified at the respective genomic loci of 12 out of 16 ETV6/RUNX1 associated lncRNAs identified above (Supplementary Figure S1). As a representative example, sequencing tracks for *lnc-LRP8-3* are shown in Figure 1D. In general, broad H3K27ac binding, covering the whole lncRNA transcript, was identified in the REH cell line for *lnc-NKX2-3-1, lnc-RTN4R-1*, *lnc-GIP-1, lnc-LRP8-3*, *lnc-TCF12-2, lnc-C8orf4-1, lnc-C8orf4-2, lnc-TINAGL1-1* and *lnc-LSM11-4*, whereas more discrete H3K27ac peaks were identified at putative promoter regions for *lnc-TIMM21-5* and *lnc-CPT2-7* (Supplementary Figure S1).

### Identification of ETV6/RUNX1 regulated lncRNAs

**Figure 2:**
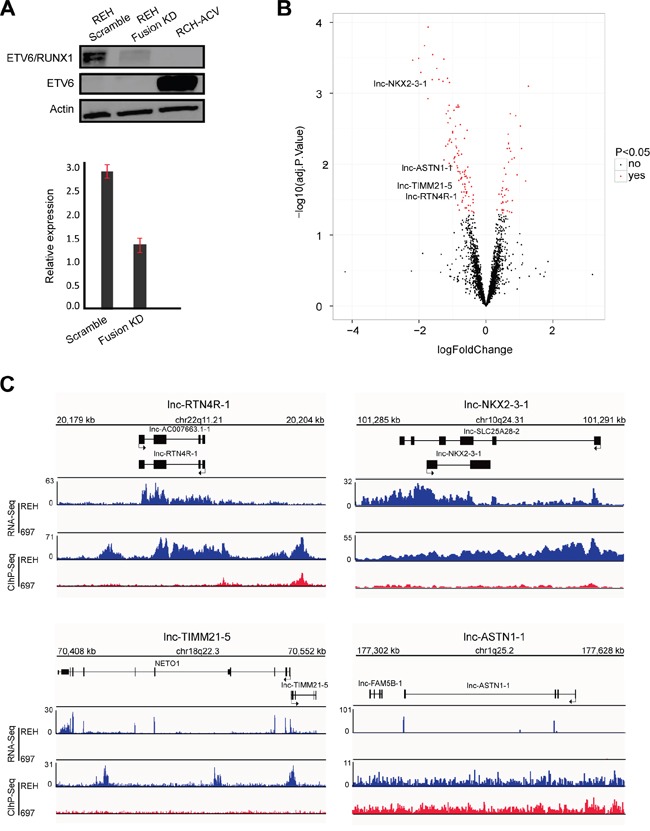
Identification of ETV6/RUNX1 regulated lncRNA **A.** Western blot (top) and RT-qPCR (below) analysis of ETV6/RUNX1 protein and transcript in REH cells upon fusion knockdown (KD). RCH-ACV used as an ETV6/RUNX1-negative B-ALL cell line with wild type ETV6. This result is representative for three independent biological replicates. **B.** Volcano plot representing the differentially expressed lncRNAs (red) when comparing fusion knockdown to the scramble (adjusted p-value < 0.05). **C.** RNA sequencing tracks (RNA-Seq) and H3K27ac binding patterns (ChIP-Seq) for *lnc-NKX2-3-1, lnc-TIMM21-5, lnc-ASTN1-1* and *lnc-RTN4R-1* in REH and 697 cell lines.

To further confirm the association between ETV6/RUNX1 and lncRNA expression signatures, we silenced the fusion protein in REH cells using a short hairpin RNA (shRNA) targeting the ETV6 moiety of the fusion transcript (Figure 2A). Given that REH cells harbor an ETV6/RUNX1 translocation with a concomitant deletion of the remaining wildtype ETV6 allele, this shRNA will exclusively modulate the expression of the fusion transcript. Next, we used the same microarray platform [26] for lncRNA profiling and identified 134 lncRNAs that showed differential expression between biological triplicates of ETV6/RUNX1 knockdown and the corresponding REH control samples (41 lncRNAs up- and 93 down-regulated; adj. p-value<0.05; Figure 2B; Supplementary Table S4).

Among the lncRNAs that were significantly affected by ETV6/RUNX1 modulation, only four transcripts (*lnc-NKX2-3-1*, *lnc-TIMM21-5*, *lnc-ASTN1-1* and *lnc-RTN4R-1*) overlapped with the 16 ETV6/RUNX1 associated lncRNAs identified above (Figure 2B). The specific expression pattern of these four lncRNAs in *ETV6/RUNX1*-positive leukemia patients as well as their H3K27ac binding patterns (ChIP-Seq) and RNA sequencing tracks in REH and 697 cells are visualized in Supplementary Figure S2 and Figure 2C, respectively.

**Figure 3:**
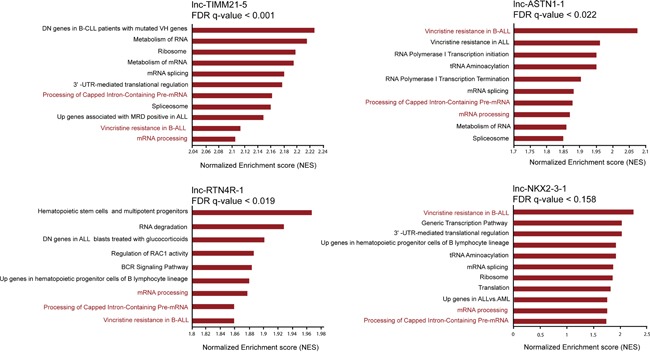
Attributing functional annotation to ETV6/RUNX1 specific lncRNAs through guilt-by-association analysis Selection of top enriched gene sets correlated with the expression of lnc-NKX2-3-1, lnc-TIMM21-5, lnc-ASTN1-1 and lnc-RTN4R-1 in 25 ETV6/RUNX1-positive BCP-ALL patients. FDR stands for false discovery rate.

To investigate potential functionalities for each of these four ETV6/RUNX1 regulated lncRNAs, we performed *in silico* guilt-by-association analysis using the lncRNA and mRNA expression profiles of 64 BCP-ALL patients. Considering that the output of this analysis is based on the correlation between the lncRNA-of-interest and all protein coding genes and the fact that these lncRNAs are ETV6/RUNX1-specific, performing this analysis in the whole cohort of patients was influenced by the subgroup bias and resulted in the same functionalities for all four lncRNAs. To remove this subgroup bias, we performed this analysis exclusively in 25 ETV6/RUNX1-positive BCP-ALL patients. Notably, this approach suggested that *lnc-NKX2-3-1*, *lnc-TIMM21-5*, *lnc-ASTN1-1* and *lnc-RTN4R-1* have both unique as well as overlapping functionalities. For example, mRNA processing, processing of capped intron-containing pre-mRNA, mRNA splicing and resistance to vincristine, were amongst the top 30 most correlated gene sets for all four lncRNAs mentioned above (Figure 3). Nevertheless, each lncRNA also showed unique patterns of positive and negative correlations at the single gene level, as shown by the top 10 most correlated transcripts for each lncRNA in Supplementary Figure S3.

Given the predicted link between these lncRNAs and resistance to vincristine, we examined whether these lncRNAs are able to identify a more aggressive subtype of *ETV6/RUNX1*-derived leukemia. We were able to compare the levels of lncRNA expression between relapsed (n=3) and non-relapsed (n=22) samples at time of diagnosis. Notably, this analysis revealed that the average expression levels of *lnc-TIMM21-5* and *lnc-RTN4R-1* are significantly higher in the diagnostic samples of the three cases that experienced relapse, as compared to the 22 *ETV6/RUNX1*-positive BCP-ALLs in complete remission (logFC=1.1; Supplementary Table S5). Moreover, we were able to collect paired samples from a new *ETV6/RUNX1*-positive ALL patient (not included in the initial 25 *ETV6/RUNX1*-positive cases), for which leukemic cells were available at the time of initial diagnosis as well as at relapse. Comparison of the expression levels of the four lncRNAs between diagnosis and relapse revealed upregulation of *lnc-NKX2-3-1*, *lnc-RTN4R-1* and *lnc-TIMM21-5* at the time of relapse, whereas *lnc-ASTN1-1* displayed an opposite behavior (Supplementary Figure S4).

### LncRNA modulation in ETV6/RUNX1-positive BCP-ALL cell lines

**Figure 4:**
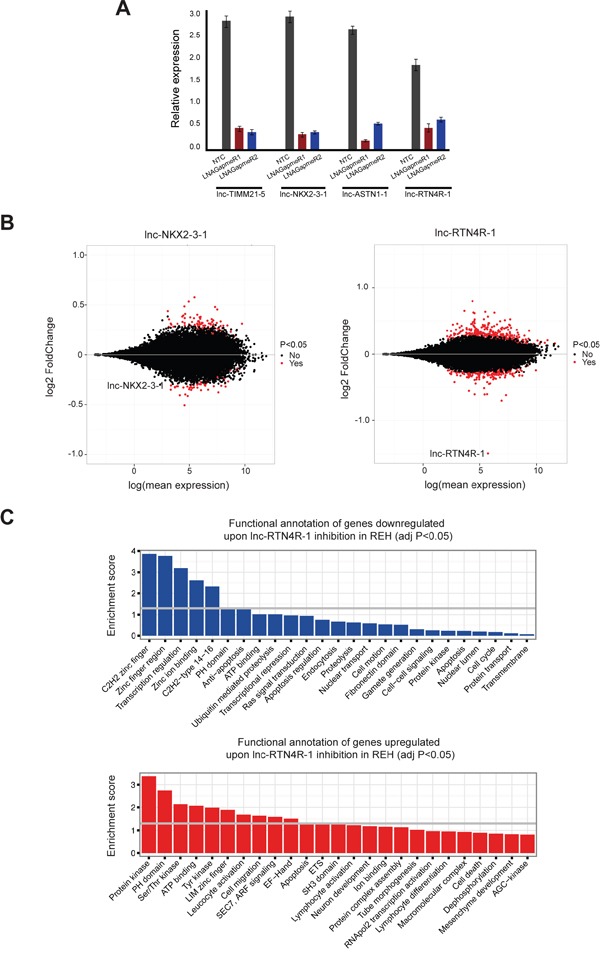
Transcriptional consequences of lncRNA modulation in the context of ETV6/RUNX1 rearranged leukemia cases **A.** RT-qPCR analysis of lncRNA knockdown experiments using two independent LNA GapmeRs for each lncRNA. This plot is representative for three independent biological replicates and knockdown was evaluated with three different primer pairs for each lncRNA. **B.** MA-plots, which display the mean expression and fold change of RNA sequencing data of REH cells upon *lnc-NKX2-3-1* and *lnc-RTN4R-1* knockdown. Significantly differentially expressed genes are represented in red. **C.** Functional annotation of genes down- and up-regulated upon lnc-RTN4R-1 knockdown in REH cell line.

To further study the biological functions of *lnc-NKX2-3*, *lnc-TIMM21*, *lnc-ASTN1* and *lnc-RTN4R* in the context of ETV6/RUNX1-driven leukemogenesis, we used LNA GapmeR technology to evaluate transcriptional effects of lncRNA modulation *in vitro*. For each lncRNA, we evaluated the capacity of eight different LNA GapmeRs to silence lncRNA expression levels in REH cells through gymnosis [29]. Using this passive uptake approach, we identified two independent LNA GapmeRs for each lncRNA that showed at least 80% lncRNA knockdown, as evaluated by RT-qPCR (Figure 4A).

To evaluate the transcriptional consequences of these lncRNA perturbations, we performed RNA sequencing and profiled biological triplicates of two independent LNA GapmeR knockdown experiments for each lncRNA together with their respective control samples in the REH cell line (6 versus 6 for each lncRNA). Strikingly, modulation of *lnc-TIMM21-5* and *lnc-ASTN1-1* caused no or very limited effects on overall transcription in REH cells (Supplementary Figure S5). However and most notably, gene transcription was severely affected by perturbations of *lnc-NKX2-3-1* and *lnc-RTN4R-1* (Figure 4B).

More specifically, *lnc-NKX2-3-1* inactivation resulted in significant changes in the expression of 75 transcripts (43 up- and 32 down-regulated; adj. p-value<0.05; Figure 4B; Supplementary Table S6). Of note, different immunoglobulin heavy and lambda transcripts (*IGHV3-15*, *IGLV2-23*, *IGLV3-10*, *IGLV3-21*, *IGLC3*, *IGLC6* and *IGLC7*) were amongst the most significantly up-regulated genes after *lnc-NKX2-3-1* modulation (Supplementary Table S6). Moreover, functional annotations of up- and down-regulated genes upon *lnc-NKX2-3-1* knockdown in REH cells revealed that this lncRNA mainly impacts transcriptional regulation (Supplementary Figure S6).

Interestingly, expression changes were even more pronounced upon *lnc-RTN4R-1* knockdown in REH cells, with significant changes for 367 transcripts (241 up- and 126 down-regulated; adj. p-value<0.05; Figure 4B; Supplementary Table S7). Of note, 23 out of the 126 down-regulated genes showed correlation (r>0.5) with *lnc-RTN4R-1* in the guilt-by-association analysis, which was performed on *ETV6/RUNX1*-positive patients samples. These data further confirm the positive regulatory role of *lnc-RTN4R-1* at transcriptional level. Moreover, functional annotation of genes down-regulated after *lnc-RTN4R-1* knockdown in REH cells revealed significant enrichment of genes involved in transcriptional regulation and anti-apoptosis (Figure 4C). In contrast, up-regulated genes upon *lnc-RTN4R-1* knockdown were enriched in kinase signalling and apoptosis (Figure 4C).

**Figure 5:**
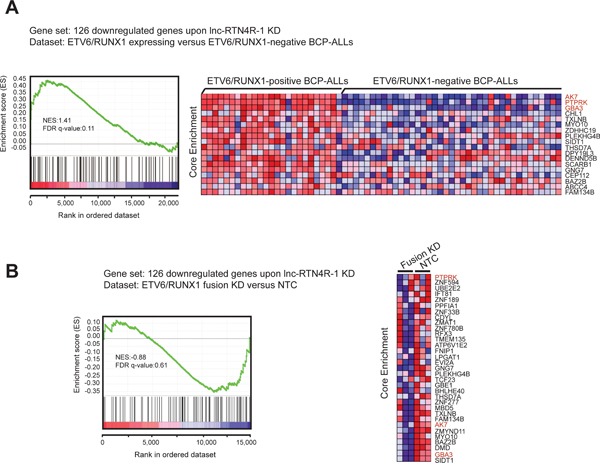
Integration of transcriptional consequences of lncRTN4R-1 modulation with ETV6/RUNX1 specific mRNA signatures in primary B-ALLs and REH cells GSEA shows that the 126 genes that are significantly down-regulated upon *lnc-RTN4R-1* knockdown are significantly enriched in both *ETV6/RUNX1*-positive BCP-ALL patient samples **A.** as well as in genes that are down-regulated upon ETV6/RUNX1 knockdown in REH cells **B.**

Finally, we evaluated to what extent the genes affected by *lnc-RTN4R-1* perturbation could also be recapitulated in the ETV6/RUNX1 specific mRNA expression profile in primary patient samples and/or the ETV6/RUNX1 knockdown signature in REH cells. Notably, GSEA analysis revealed significant enrichment of genes down regulated upon *lnc-RTN4R-1* knockdown in transcripts that are highly expressed in *ETV6/RUNX1*-positive BCP-ALL, suggesting that *lnc-RTN4R-1* indeed plays a role in the establishment of part of the *ETV6/RUNX1*-specific gene expression signature (Figure 5A). More specifically, from the 126 transcripts that are significantly down regulated upon *lnc-RTN4R-1* knockdown in REH cells, nine were significantly up regulated in *ETV6/RUNX1*-positive BCP-ALLs. The leading edge of this analysis included AK7, PTPRK and GBA3, which are the most prominent examples of genes that are significantly down-regulated after lnc-RTN4R-1 knockdown, highly expressed in ETV6/RUNX1-positive BCP-ALL and also significantly down-regulated after fusion protein knockdown (Figure 5A–5B).

## DISCUSSION

Emerging evidence suggests that lncRNAs are critically involved in a variety of human tumor entities [20–23] and the identification of cancer-associated lncRNAs might reveal new prognostic biomarkers or novel therapeutic strategies for the treatment of human cancer. In this study, we characterized the lncRNA expression signature associated with *ETV6/RUNX1-positive* BCP-ALL, one of the most prevalent genetic subtypes of childhood leukemia.

A previous study by Fernando and colleagues [24] also evaluated lncRNA expression signatures in genetic subtypes of human BCP-ALL using 14 *ETV6/RUNX1*, 15 *TCF3/PBX1* and 15 MLL-rearranged leukemia specimens. Although their study was not only focused on the identification of subtype specific lncRNA signatures, the ETV6/RUNX1 specific expression of *lnc-BTBD10-3* (*LINC00958*) reported in their study, could be confirmed in our dataset.

However, besides using primary BCP-ALL patient samples, we also integrated these lncRNA expression data with RNA sequencing results from a panel of human BCP-ALL leukemia cell lines and identified a unique lncRNA expression profile of 16 lncRNAs exclusively associated with the presence of the ETV6/RUNX1 fusion protein. Notably, *lnc-SARDH-1* (also known as *DBH-AS1*) was the only lncRNA from this list for which an oncogenic role has previously been postulated. Indeed, *DBH-AS1* promotes cell proliferation and cell survival through activation of MAPK signaling in the context of hepatocellular carcinoma [30].

Given that previous studies revealed putative overlap between lncRNA expression and regulatory enhancer elements [31–33], we also evaluated the distribution of H3K27ac, a histone modification associated with enhancer activity, at the genomic loci of the 16 ETV6/RUNX1 specific lncRNAs mentioned above. Notably, broad H3K27ac binding was identified for *lnc-NKX2-3-1, lnc-RTN4R-1*, *lnc-GIP-1, lnc-LRP8-3*, *lnc-TCF12-2, lnc-C8orf4-1, lnc-C8orf4-2, lnc-TINAGL1-1* and *lnc-LSM11-4* in *ETV6/RUNX1*-positive REH cells. Interestingly, coding genes adjacent to some of these lncRNAs showed unique overexpression in *ETV6/RUNX1*-positive BCP-ALLs, suggesting a possible *cis* regulatory relationship between these lncRNAs and their nearby protein coding genes. For example, the RNA-binding protein *IGF2BP1*, which is one of the most significantly up-regulated mRNA transcripts in *ETV6/RUNX1*-positive BCP-ALL and acts as a potent regulator of ETV6/RUNX1 mRNA stability [34], is located antisense of *lnc-GIP-1* with an overlap at their respective first exons. Therefore, *lnc-GIP-1* could be directly involved in the regulation of *IGF2BP1* expression in the pathogenesis of BCP-ALL. Notably, *IGF2BP1* has recently been reported as a novel IGH translocation partner in BCP-ALL [35]. Furthermore, we also identified a strong correlation in *ETV6/RUNX1*-positive leukemias between *NETO1* and its adjacent, bidirectional lncRNA *lnc-TIMM21-5* (r = 0.89) as well as between *LRP8* and *lnc-CPT2-7* (r = 0.70). Of note, *lnc-TIMM21-5* and *lnc-CPT2-7* showed discrete H3K27ac peaks at their respective promoter sites, instead of broad H3K27ac binding as was observed for some of the other lncRNAs. It will be of interest to investigate whether these lncRNAs truly participate in the local gene regulation. However, a recent study showed that correlation between expression of a lncRNA and its neighboring protein coding gene might be due to the processes of lncRNA's transcription or splicing which increase local concentration of transcription-associated factors at the promoter loci of adjacent protein coding gene regardless of the lncRNA sequence [36].

Finally, we applied shRNA-mediated silencing of endogenous ETV6/RUNX1 to narrow down the list of the lncRNAs are truly regulated by the oncogenic fusion protein to *lnc-NKX2-3-1, lnc-TIMM21-5, lnc-ASTN1-1* and *lnc-RTN4R-1*. Interestingly, subsequent lncRNA modulation experiments using LNA GapmeR technology revealed that *lnc-TIMM21-5* and *lnc-ASTN1-1* modulation has no effect on overall transcription, suggesting that these lncRNAs might act at the translational level, as has been described for other lncRNAs, including the PU.1 antisense lncRNA [37] or/and at various processing steps of pre-mRNA. In contrast, *lnc-NKX2-3-1* and *lnc-RTN4R-1* perturbations resulted in severe changes in gene expression, suggesting an alternative mechanism of action for these lncRNAs that is more transcriptionally oriented. Most notably, loss of *lnc-RTN4R-1* expression significantly affected part of the *ETV6/RUNX1*-specific mRNA expression signature as exemplified by reduced levels of *AK7*, *PTPRK* and *GBA3*. All together, our study identified a panel of *ETV6/RUNX1*-specific lncRNAs that might be implicated in the biology of human BCP-ALL and could serve as novel therapeutic target for this prevalent subtype of human leukemia.

## MATERIALS AND METHODS

### Clinical samples

Diagnostic bone marrow samples of 64 children with BCP-ALL, enrolled in subsequent trials of the European Organization for Research and Treatment of Cancer, Childhood Leukemia Group (EORTC-CLG) between July 1998 to July 2012, were acquired after obtaining informed consent from patients or their legal guardians according to the declaration of Helsinki. This cohort consisted of 25 cases with t(12;21)(p13;q22) [ETV6/RUNX1], seven cases with t(1;19)(q23;p13) [TCF3/PBX1], 15 high hyperdiploid BCP-ALLs (>50 chromosomes) and 17 BCP-ALLs with a normal karyotype (absence of any numerical aberrations or translocations). Moreover, bone marrow samples at the time of initial diagnosis (1998-2012) as well as at relapse (2015) were acquired from a new ETV6/RUNX1 patient, which was not included in the initial 25 ETV6/RUNX1 cases. High-quality RNA from the bone marrow samples was isolated and was used for microarray based expression profiling.

### Cell culture

BCP-ALL cell lines (REH, RCH-ACV, 697) were obtained from the DSMZ (Deutsche Sammlung von Mikroorganismen und Zellkulturen GmbH) repository. Cells were cultured in RPMI 1640 media (Life Technologies Europe) supplemented with 10% fetal bovine serum (Biochrom AG), 1% penicillin/streptomycin (Life Technologies Europe), 1% kanamycin (Life Technologies Europe), 1% glutamine (Life Technologies Europe) at 37°C in 5% CO2.

### RNA isolation, cDNA synthesis and RT-qPCR

Total RNA was isolated using the miRNeasy mini or micro kit (Qiagen) according to the manufacturer's instructions. For each sample, RNA quality and purity were assessed by Experion analysis (Bio-Rad, Nazareth Eke, Belgium) and concentration was measured using the NanoDrop ND-1000 (NanoDrop Technologies, Wilmington, DE, USA). cDNA was generated using the iScript cDNA synthesis kit (Bio-Rad, Nazareth Eke, Belgium) according to the instructions of the manufacturer. After cDNA preparation, reverse transcription quantitative real-time polymerase chain reaction (RT-qPCR) was carried out using custom 2X SsoAdvanced SYBR Green Supermix (Bio-Rad) in two technical replicates. Briefly, 2 μl cDNA (2.5 ng/μL) was added to 3 μl of PCR mix (2.5 μl 2X SsoAdvanced mastermix, 0.25 μl Forward Primer (5μM), 0.25 μl Reverse Primer (5μM)). qRT-PCR was performed on a LightCycler 480 (Roche) and data were analyzed using qBasePLUS software (Biogazelle) [38]. Relative expression values were calculated using the ΔCt-method. For normalization, the expression of at least three reference genes was combined to calculate a normalization factor. The primer sequences are listed in Supplementary Table S8.

### Western blotting

Total protein isolation was performed using RIPA-lysis buffer, supplemented with protease inhibitors and SDS-PAGE was executed according to standard protocols. Blotting was performed on a PVDF Membrane and the membrane blocked with 5% milk. For immunoblotting, the mouse monoclonal antibody against ETV6 (Abnova, H00002120-M01) was used in a 1:1000 dilution and the mouse monoclonal antibody against actin (Sigma, A2228) in a 1:5000 dilution. The anti-mouse secondary antibodies were used in a 1:5000 dilution.

### Microarray profiling

RNA samples were profiled on a custom designed Agilent micro-array covering all protein coding genes and 23042 lncRNAs as described by Volders et al. [26]. A total of 100 ng input RNA was used to complete the profiling according to the manufacturer's protocol (One-color Microarray-Based Gene Expression Analysis, Low Input Quick Amp Labeling, Agilent Technologies). Normalization of the expression data was performed using the VSN-package (BioConductor release 2.12) in R bioconductor. Expression values were further subjected to background subtraction and probes detecting a 10% higher expression as compared to the negative controls in at least 60% of one genetic subtype of BCP-ALL, were retained for further analysis. Following the normalization of gene-expression microarray data and background correction, differentially expressed genes between *ETV6/RUNX1*-positive BCP-ALLs and other subgroups were determined using the limma in the R bioconductor. All p-values were corrected for multiple testing using the ‘‘Benjamini-Hochberg’’ correction method (p<0.05). Data were submitted to Gene Expression Omnibus (GSE79873).

### RNA-seq analysis

Publically available RNA sequencing data were obtained from the Cancer Cell Line Encyclopedia for a panel of 13 human BCP-ALL cell lines including one *ETV6/RUNX1*-positive (REH) and 12 *ETV6/RUNX1*-negative (SEM, SUP-B15, NALM-6, NALM-19, RCH-ACV, RS4;11, KASUMI-2, KOPN-8, MUTZ-5, MHH-CALL-2, MHH-CALL-3, MHH-CALL-4) human BCP-ALL cell lines.

Total RNA was extracted from REH and 697 cells, as well as from REH cells treated with LNA gapmeRs against four different lncRNAs and a non-targeting gapmeR using the miRNeasy mini kit (Qiagen) according to the manufacturer's instructions. The RNA quality was evaluated using a RNA Nano chip on a Bioanalyzer (Agilent technologies). For library preparation, the Illumina TruSeq RNA sample Prep Kit (San Diego, CA, USA) was used according to the manufacturer's protocol. Library quality was checked using a DNA-1000 chip on a Bioanalyzer (Agilent technologies). Briefly, 100 ng of total RNA was poly-A purified, fragmented and first-strand cDNA reverse transcribed using the Truseq protocol. Next, second-strand cDNA synthesis, end repair, addition of a single A base, adaptor ligation and PCR amplification was performed and enriched cDNA libraries were sequenced using an Illumina NextSeq 500 sequencer instrument. RNA sequencing data have been deposited to the GEO repository (GSE79873). Reads were aligned to the reference genome hg38 with STAR-2.4.2a and differential expression analysis was performed using DESeq2 [28].

### Lentiviral short hairpin RNA knockdown of the ETV6/RUNX1 fusion

Two ETV6 short hairpin (sh) (TRCN0000003854, TRCN0000003855) were purchased from Sigma-Aldrich and the green fluorescent protein (GFP) gene was inserted in the backbone of these vectors. Only one of these ETV6 specific shRNAs targeted the ETV6/RUNX1 fusion transcript, whereas the other was used as a non-targeting control. These plasmids were co-transfected with packaging and envelope vectors in HEK293TN cells using jet-PEI (VWR). 48h following transfection, viral supernatants were collected and REH cells were transduced in the presence of 8 μg/mL polybrene by spinoculation at 2300 rpm, 32°C for 90 minutes. After 72 hours, GFP-positive cells were sorted using the Bio-Rad S3e Cell Sorter device.

### LNA treatment of BCP-ALL cell lines

REH cells were seeded in 48-well plates (260μl, 300,000 cells/well) and transfected with LNA™ (locked nucleic acid) GapmeRs (Exiqon) by gymnosis (passive uptake) to obtain a final GapmeR concentration of 10μM (Supplementary Table S9). Next, RNA isolation was carried out using miRNeasy micro kit (Qiagen) according to the manufacturer's instructions 72h after transfection. The biological triplicates for these experiments were executed on different days.

### Chromatin immunoprecipitation (ChIP) sequencing

ChIP sequencing was performed as previously described [39] starting from 10*10^7^ REH cells. Nuclei were isolated and chromatin was purified by chemical lysis and the purified chromatin was fragmented to 200-300 bp fragments by sonication (Covaris, S220, Focused-ultrasonicator). Chromatin immunoprecipitation was performed by incubation of the chromatin fraction overnight with 100 μl of protein-A coated beads (Thermo-Scientific) and 10 μg of fibrillarin-specific (Abcam, ab5821) or H3K27ac-specific antibodies (Abcam, ab4729). The next day, beads were washed to remove non-specific binding events and enriched chromatin fragments were eluted from the beads, followed by reverse cross-linking by incubation at 65°C overnight. DNA was subsequently purified by phenol/chloroform extraction and used for library preparation and sequencing using an Illumina Hiseq 2000 device (San Diego, CA, USA). Raw sequencing data were mapped to the human reference genome (GRCh37/h19) using Bowtie. Peak calling was performed using MACS 1.4. The ChIP-seq data were deposited in the GEO database (GSE79873).

### Guilt-by-association analysis

Spearman correlation coefficients were calculated between lncRNAs and all protein coding genes using expression data of BCP-ALLs profiled on the custom designed Agilent array. The correlation values of each lncRNA and all protein coding genes were subsequently ranked from the highest to the lowest value and used as input for pre-ranked gene set enrichment analysis (GSEA), using the c2v5.0 MsigDB collection as gene set database (http://www.broad.mit.edu/gsea/) [40].

## SUPPLEMENTARY MATERIALS TABLES FIGURES




















