# Supplementary material for: Unique long non-coding RNA expression signature in ETV6/RUNX1-driven B-cell precursor acute lymphoblastic leukemia
Source: Oncotarget. 2016 Sep 16;7(45):73769–80. doi: 10.18632/oncotarget.12063 (PMC5342012; doi:10.18632/oncotarget.12063)
Supplement: Supplementary file 1 [file oncotarget-07-73769-s001.pdf]

Unique long non-coding RNA expression signature in ETV6/ RUNX1-driven B-cell precursor acute lymphoblastic leukemia

SUPPLEMENTARY FIGURES AND TABLES

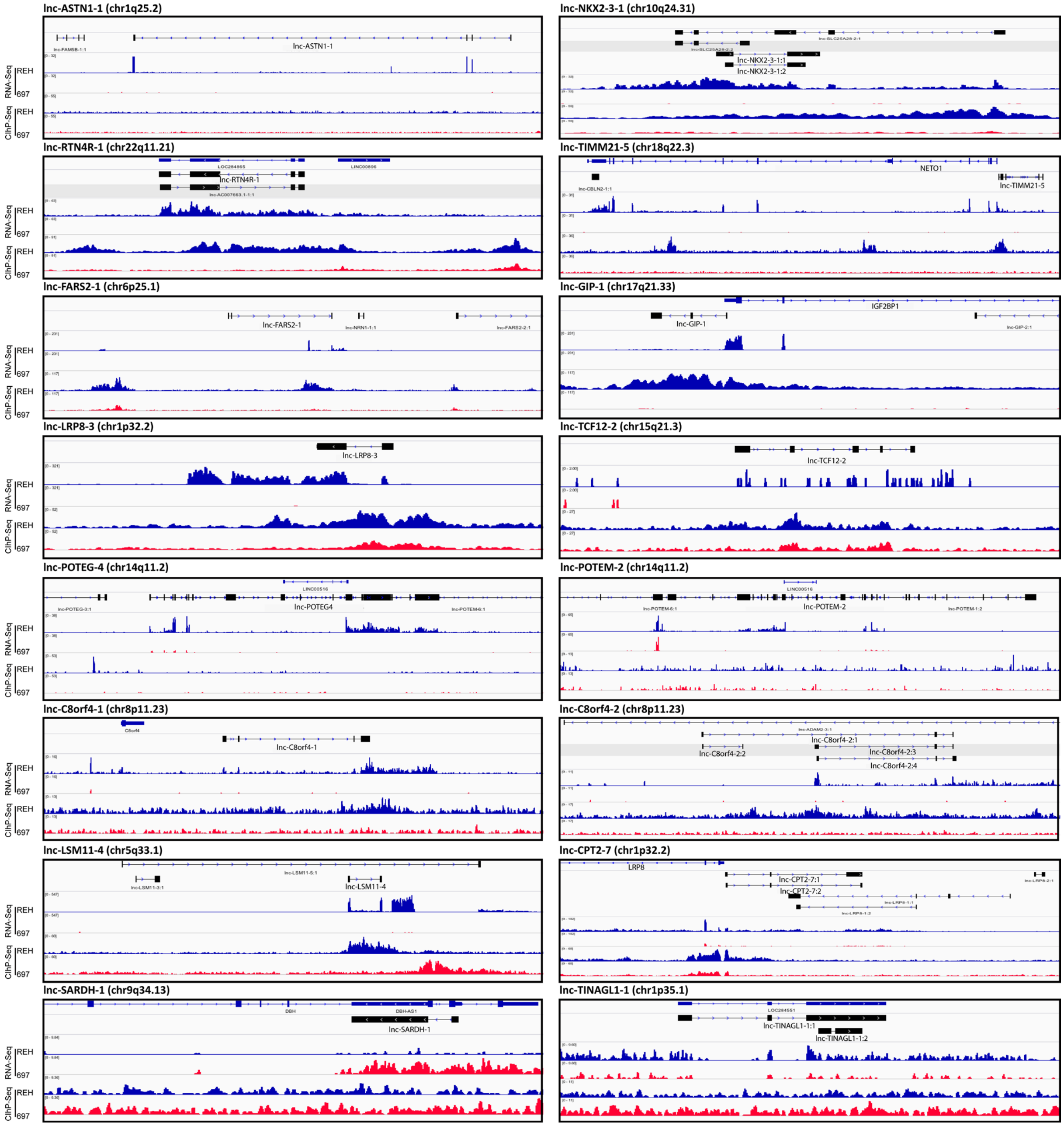

Supplementary Figure S1: RNA sequencing tracks and H3K27ac binding patterns of all 16 lncRNAs in REH and 697 cell lines.

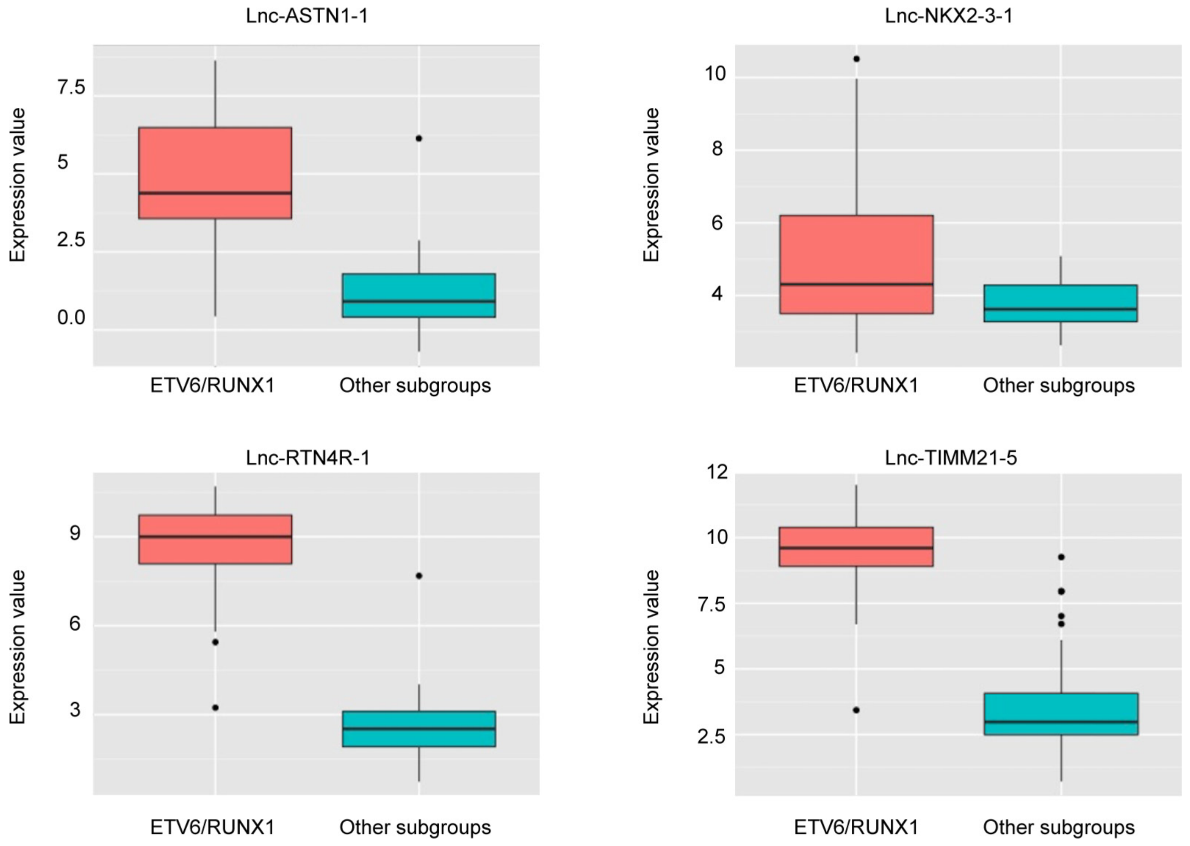

Supplementary Figure S2: The expression level of *lnc-NKX2-3-1*, *lnc-TIMM21-5*, *lnc-ASTN1-1* and *lnc-RTN4R-1* in *ETV6/RUNX1*-positive BCP-ALLs *versus* other BCP-ALL subgroups.

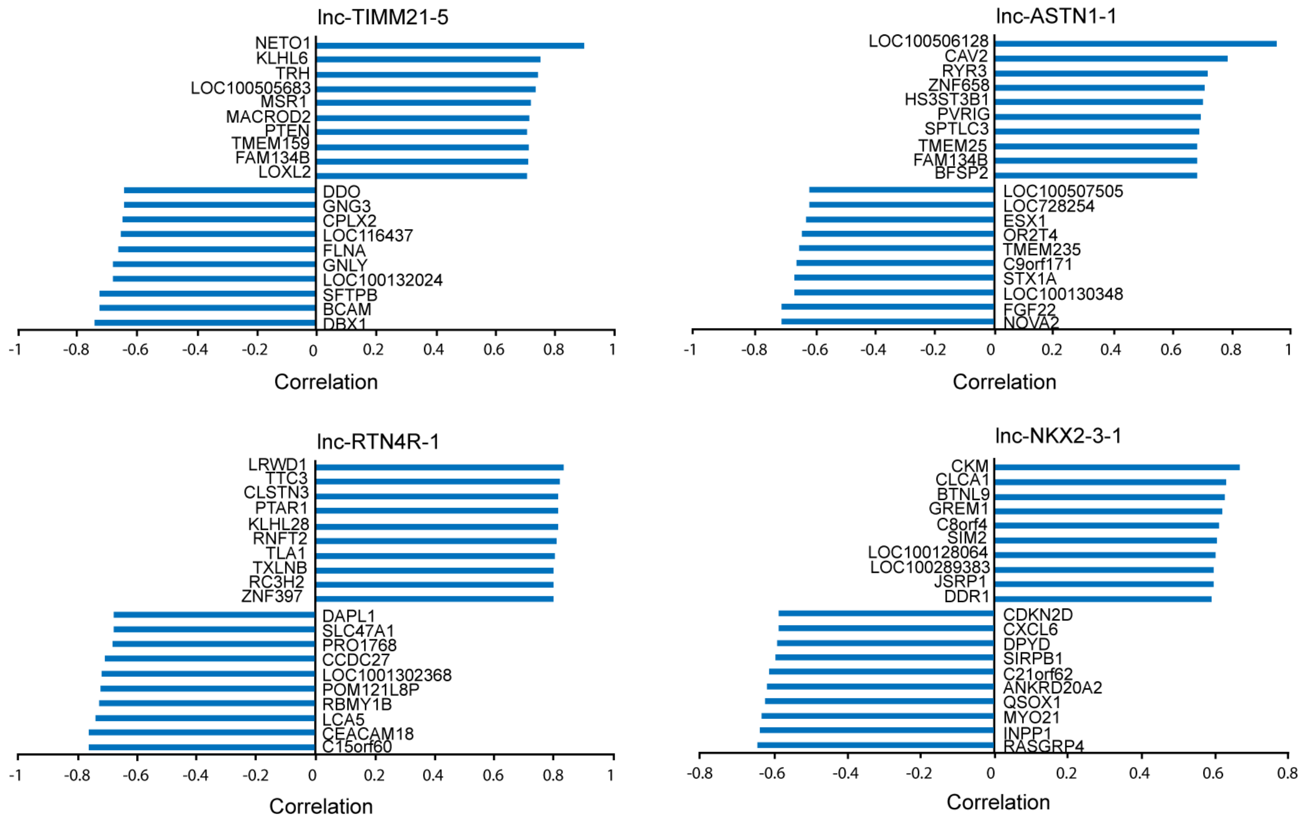

Supplementary Figure S3: Top 10 transcripts that are most positively and negatively correlated with the expression of *lnc-NKX2-3-1*, *lnc-TIMM21-5*, *lnc-ASTN1-1* and *lnc-RTN4R-1* in 25 ETV6/RUNX1-positive BCP-ALLs.

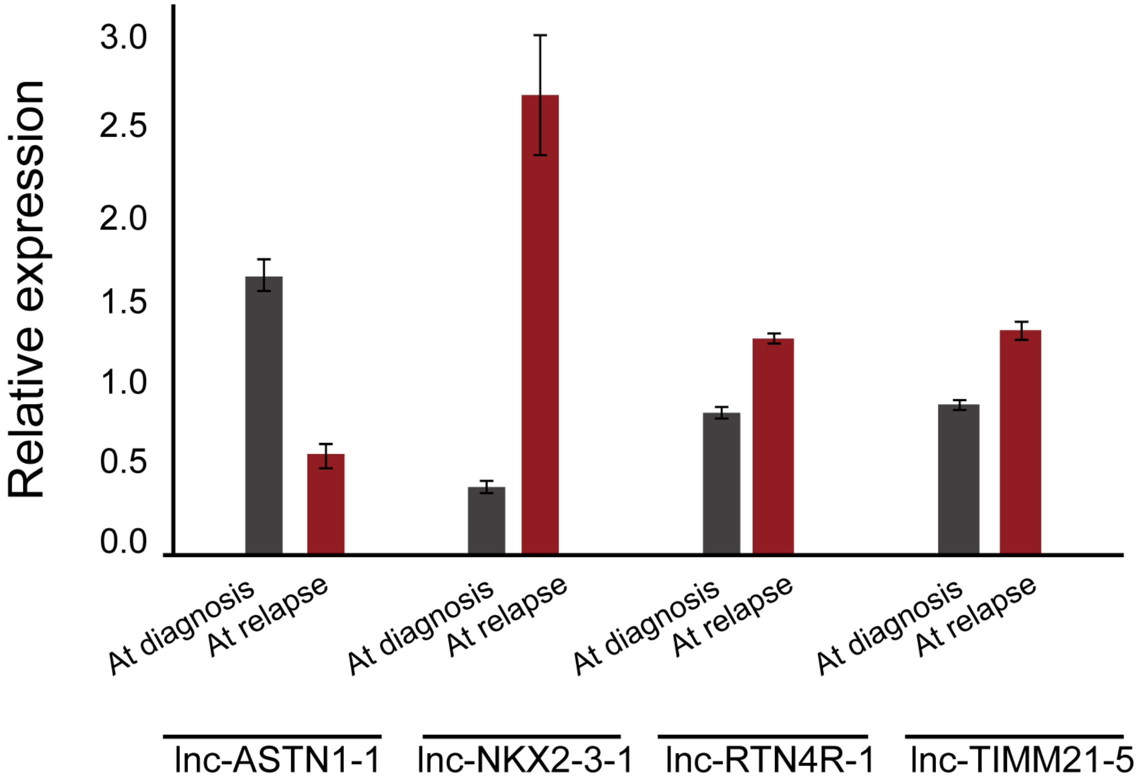

Supplementary Figure S4: Comparison of expression level of *lnc-NKX2-3-1*, *lnc-TIMM21-5*, *lnc-ASTN1-1* and *lnc-RTN4R-1* between diagnosis and relapse samples of an *ETV6/RUNX1*-positive case.

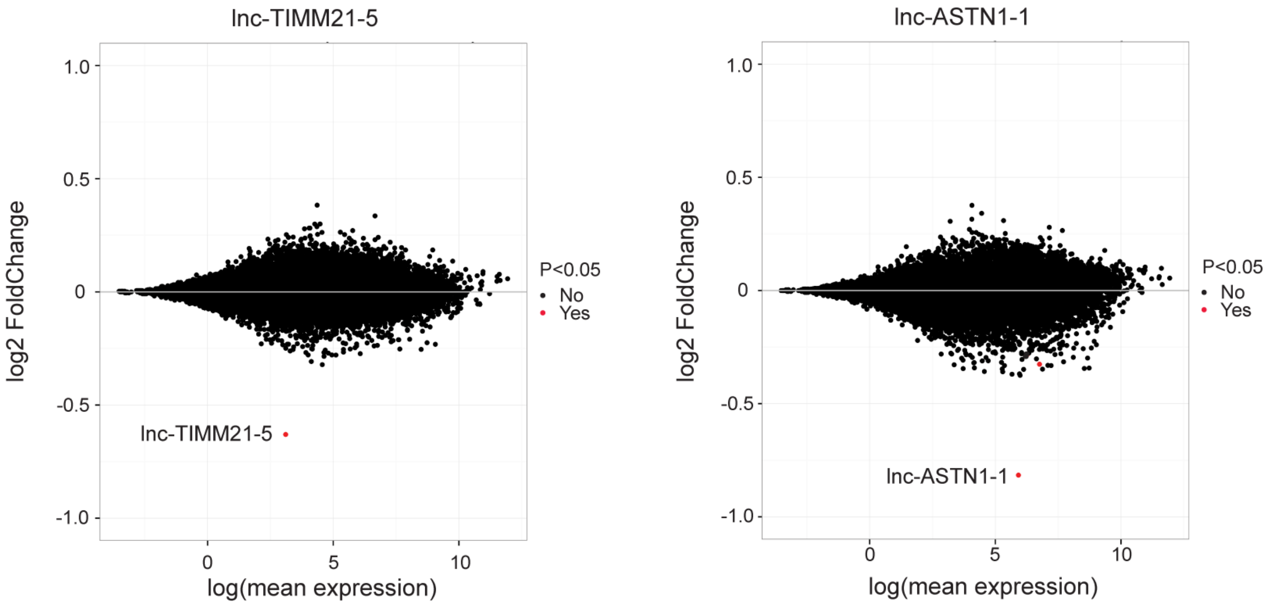

Supplementary Figure S5: MA-plots, which display the mean expression and fold change of RNA sequencing data of REH cells upon *lnc-TIMM21-5* and *lnc-ASTN1-1* knockdown.

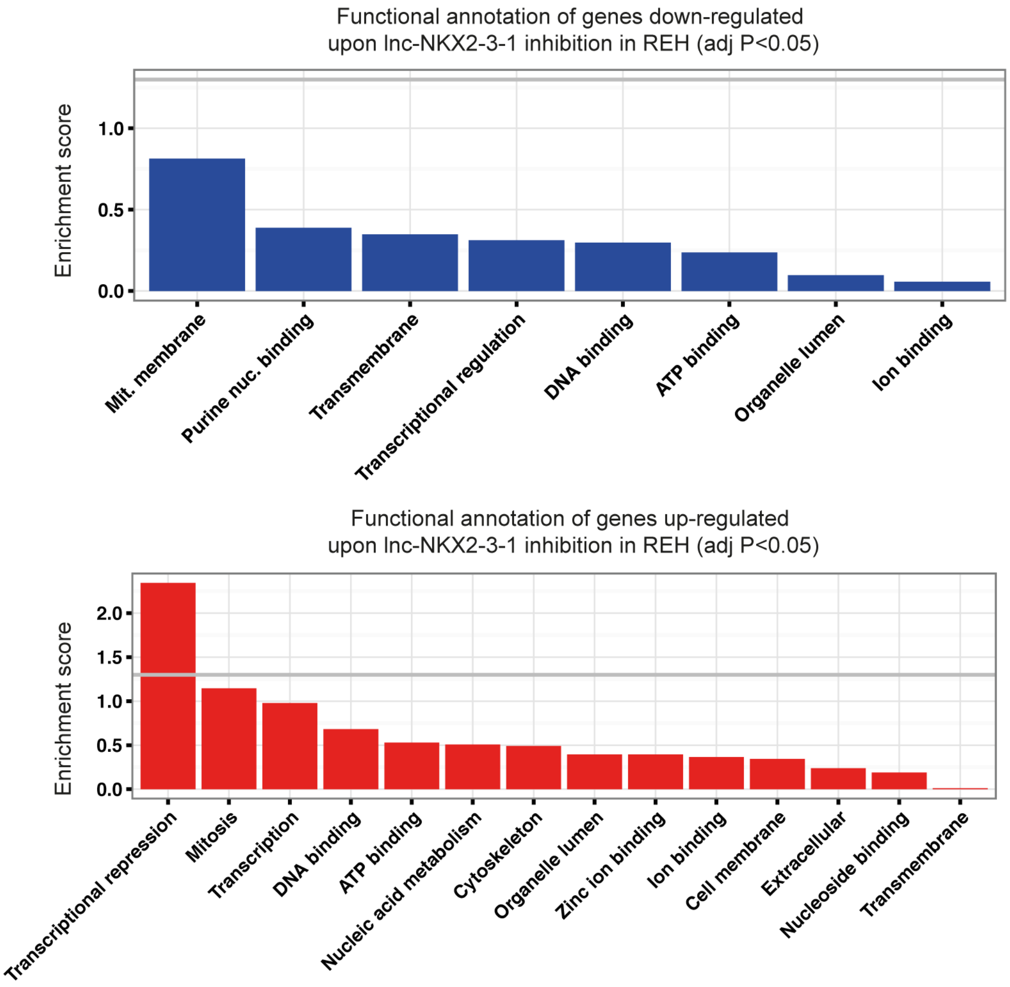

Supplementary Figure S6: Functional annotation of down- and up-regulated genes upon lnc-NKX2-3-1 knockdown in REH cell line.

Supplementary Table S1: Significantly differentially expressed lncRNAs in ETV6/RUNX1-driven BCP-ALLs.

See Supplementary File 1

Supplementary Table S2: Significantly differentially expressed lncRNAs in REH cell line comparing other B-ALL cell lines revealed by RNA-sequencing.

See Supplementary File 2

Supplementary Table S3: Significantly differentially expressed lncRNAs in ETV6/RUNX1-positive BCP-ALLs and B-ALL cell line.

See Supplementary File 3

Supplementary Table S4: Significantly differentially expressed lncRNAs upon the ETV6/RUNX1 knockdown in REH cell line.

See Supplementary File 4

Supplementary Table S5: The levels of four lncRNA expression in relapsed and non-relapsed samples at time of diagnosis.

See Supplementary File 5

Supplementary Table S6: Significantlly differentially expressed genes upon lnc-NKX2-3-1 knockdown.

See Supplementary File 6

Supplementary Table S7: Significantlly differentially expressed genes upon lnc-RTN4R-1 knockdown.

See Supplementary File 7

Supplementary Table S8: Primers used for ETV6/RUNX1 and lncRNAs knockdown evaluation.

See Supplementary File 8

Supplementary Table S9: LNA GapmeRs sequence used for lncRNAs knockdown.

See Supplementary File 9
